# Shifting material source of Chinese loess since ~2.7 Ma reflected by Sr isotopic composition

**DOI:** 10.1038/srep10235

**Published:** 2015-05-21

**Authors:** Wenfang Zhang, Jun Chen, Gaojun Li

**Affiliations:** 1 MOE Key Laboratory of Surficial Geochemistry, Department of Earth Sciences, Nanjing University, 163 Xianlindadao, Nanjing 210046, China

## Abstract

Deciphering the sources of eolian dust on the Chinese Loess Plateau (CLP) is fundamental to reconstruct paleo-wind patterns and paleo-environmental changes. Existing datasets show contradictory source evolutions of eolian dust on the CLP, both on orbital and tectonic timescales. Here, the silicate Sr and Nd isotopic compositions of a restricted grain size fraction (28–45 μm) were measured to trace the source evolution of the CLP since ~2.7 Ma. Our results revealed an unchanged source on orbital timescales but a gradual source shift from the Qilian Mountains to the Gobi Altay Mountains during the past 2.7 Ma. Both tectonic uplift and climate change may have played important roles for this shift. The later uplift of the Gobi Altay Mountains relative to the Qilian Mountains since 5 ± 3 Ma might be responsible for the increasing contribution of Gobi materials to the source deserts in Alxa arid lands. Enhanced winter monsoon may also facilitate transportation of Gobi materials from the Alxa arid lands to the CLP. The shifting source of Asian dust was also reflected in north Pacific sediments. The finding of this shifting source calls for caution when interpreting the long-term climate changes based on the source-sensitive proxies of the eolian deposits.

The eolian deposits on the Chinese Loess Plateau (CLP) provide a valuable archive for paleo-environmental changes^1^,^2^. The CLP began to receive massive atmospheric dust since at least the late Oligocene^3^,^4^, which has been affected by three notably prominent exogenic processes of the late Cenozoic, namely the uplift of Tibetan Plateau, the Cenozoic cooling, and the retreat of the Paratethys^5^,^6^,^7^. Many of paleo-proxies have been developed assuming an unchanged source region^8^,^9^,^10^. Thus, source research is crucial to understand the paleo-proxies developed for the loess deposits on the CLP.

The radiogenic isotopic tracers, such as Nd, Sr and Pb isotopes, have been widely used to trace the source of eolian dust on the CLP and its response to the tectonic and climatic oscillations^11^,^12^,^13^,^14^,^15^. Combined with other mineral and geochemical tracers^16^,^17^,^18^,^19^, a similar conclusion seems to be reached in recent years that Alxa arid lands, which receive materials mainly from the Gobi Altay Mountains to its north and the Qilian Mountains to its south through fluvial systems^13^, are the main source regions for the late-Pleistocene loess^20^. However, it is still controversial that whether the detrital source of eolian dust on the CLP changed on both orbital and tectonic timescales.

According to the unchanged Nd isotopes, Jahn *et al.*^21^ suggested no provenance shift on orbital timescales. In contrast, the electron spin resonance signal intensity and crystallinity index of fine-grained quartz and detrital zircon ages suggested that the provenance of loess and paleosol on the CLP is heterogeneous and spatially variable at least during last glacial-interglacial cycle^17^,^22^,^23^. Recently, Che and Li^19^ reported more datasets of detrital zircon ages with less statistical uncertainties; Nie and Peng^24^ conducted detailed studies on the assemblage of heavy minerals. Both of the two studies indicated no glacial-interglacial and spatial change in eolian source for the loess on the CLP. These controversies may be originated from the different size fractions of dust particles on the CLP, as the electron spin resonance signal intensity and crystallinity index are based on fine-grained quartz, while detrital zircon age distributions and heavy minerals analysis are based on the coarse particles.

On tectonic timescales, the shifting Sr, Nd, and Pb isotopic compositions of the <20 μm silicate fractions at the boundary of loess-paleosol and red clay indicated a source shift possibly in response to the gradual additions of relatively young orogenic materials by glacial grinding in central Asia^15^,^25^. However, Wang *et al.*^26^ argued that the decreasing ^87^Sr/^86^Sr ratios over the past 2.5 Ma may reflect increasing grain size rather than source shift while the small changes in ε_Nd_ values might be within the external analytical error. Considering the relatively small variations of Nd isotopic composition of the source materials, the controversies on the source shift of Asian dust might be solved by Sr isotopic composition when the influence of grain size and pedogenic alternation on the ^87^Sr/^86^Sr ratio are carefully considered.

The Sr isotopic composition of sediments can be strongly dependent on the grain size distribution and chemical weathering^27^,^28^. The influence of grain size on the ^87^Sr/^86^Sr ratio may be excluded by using restricted grain size fraction. Previous investigation indicates that the grain size effect is mainly contributed by the clay minerals in the <2 μm size fraction^28^. The <2 μm clay fraction has much higher ^87^Sr/^86^Sr ratio than the >2 μm fractions while the >2 μm fractions have very similar ^87^Sr/^86^Sr ratios due to the limited changes in the content of clay minerals^28^. Thus, the usage of <20 μm size fraction in tracing dust sources^25^ may exaggerate the influence of grain size change on the ^87^Sr/^86^Sr ratio. Recently, Chen and Li^14^ used the silicate Sr isotopic compositions of a specific grain size (28–45 μm) fraction as a sensitive source tracer. Combined with Nd isotopic composition, they concluded that this specific grain size Sr isotopic composition is mainly controlled by the source change other than eolian sorting^14^. The data of Chen and Li^14^ indicated the source shift of the CLP over the past 2.6 Ma, but the details of the source shift are still unclear due to the low-resolution data (only 10 data point).

This work provides a high-resolution (~30 thousand years per sample) silicate Sr isotopic records of the 28–45 μm grain size fraction of the eolian dust on the CLP since ~2.7 Ma. Combined with Nd isotopic data, the paper aims to constrain the source evolution of the eolian deposits on the CLP on both orbital and tectonic timescales. This work also discusses the possible source shift of Asian dust reflected in the Pacific sediments, based on the ^87^Sr/^86^Sr data of eolian dust extracted from the north Pacific sediments in previous study^29^.

## Results

**Figure 1:**
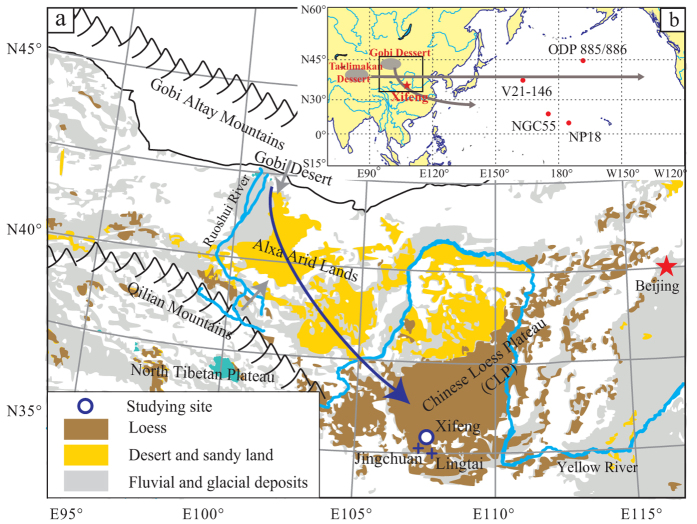
**Location map of this study. a**, Map shows the geographic setting and sampling site. Blue arrow indicates transportation of eolian dust by northwesterly wind. Gray arrows indicate input of terrigenous materials to the Alxa arid lands from the Qilian Mountains and the Gobi Altay Mountains by fluvial systems^13^. For discussion, Jingchuan site^25^ and Lingtai site^14^,^26^ with published Nd and Sr isotopes are also shown. **b**, Map shows the distributions of Gobi desert, Taklimakan desert and the locations of the Pacific cores^29^,^46^,^47^ used in this study. Arrows show transportation of Taklimakan dust and Gobi dust by westerly wind and winter monsoon, respectively. We used the “Matlab” software to generate the two maps and the maps will not have a copyright dispute.

**Figure 2:**
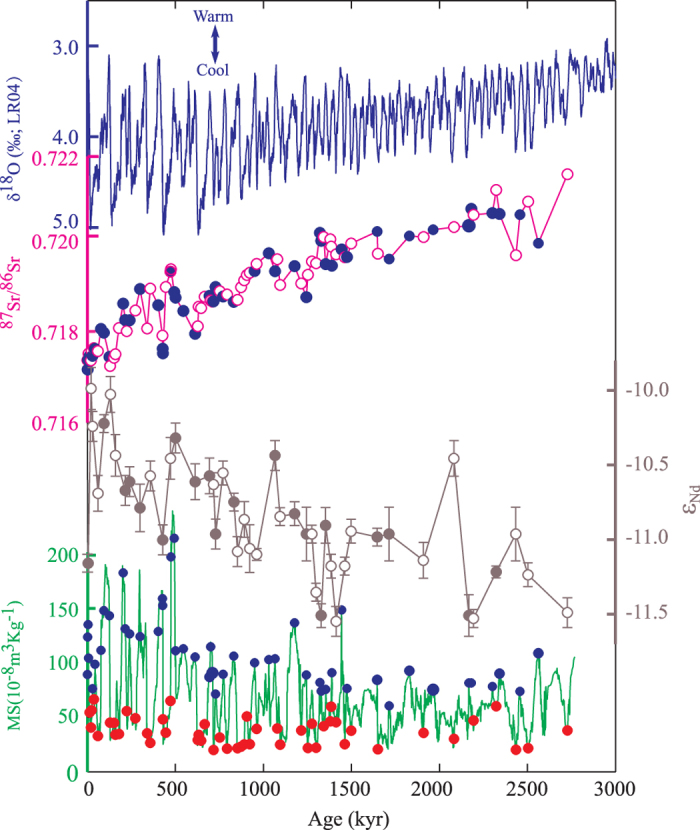
**Evolutions of Sr and Nd isotopic compositions of the eolian deposits of the Xifeng section on the Chinese Loess Plateau.** From top to bottom, are the global ice volume and/or temperature variations reflected by the oxygen isotopic composition of benthic foraminifera^36^, the evolutions of Sr (the blue solid dots are corresponding to the paleosol samples) and Nd isotopic compositions with error bars (the solid dots are corresponding to the paleosol samples) of the eolian deposits since ~2.7 Ma in this study, and the stratigraphy of magnetic susceptibility of the Xifeng section with red solid dots (loess samples) and blue solid dots (paleosol samples), showing the locations of the samples selected for Sr isotopic analysis.

**Figure 3:**
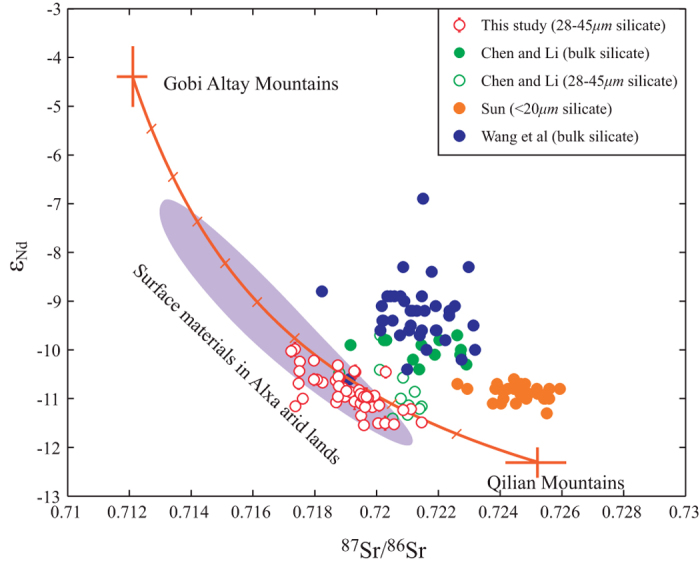
**Cross plot between the silicate Nd and Sr isotopic compositions of eolian dust on the Chinese Loess Plateau.** The average isotopic compositions of the two endmembers and isotopic ranges of the materials in the Alxa arid lands are based on <75 μm silicate fraction^11^,^13^. The mixing line is in 10% steps. Also shown are the Nd and Sr isotopic compositions of Jingchuan section^25^ and Lingtai section^14^,^26^.

**Figure 4:**
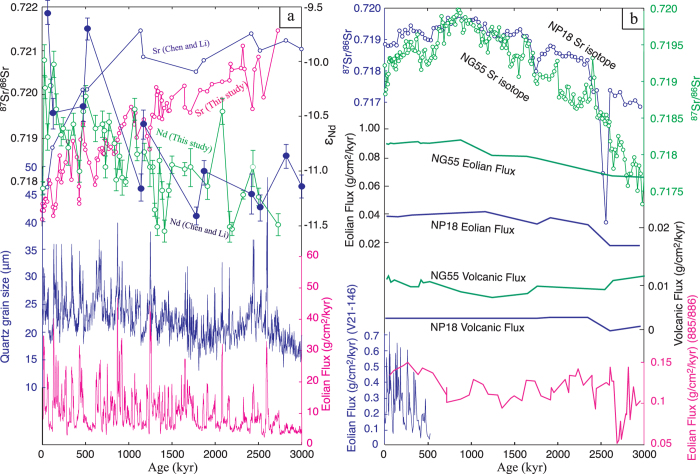
**Comparison of Sr-Nd isotopic evolution with other records of the Chinese Loess Plateau and Pacific cores. a**, Map shows the evolutions of Sr and Nd isotopic compositions of eolian deposits of the Xifeng section (this study), Lingtai section^14^ and Quartz grain size^30^,^31^ and eolian flux^38^ of the Xifeng section on the CLP; **b**, Map shows the evolutions of Sr isotopic compositions and the eolian flux and volcanic ash flux in Pacific sediments^29^, and also the eolian flux of other two Pacific sites, V21–146^46^ and 885/885^47^.

Samples for Sr and Nd analysis were collected from the Xifeng site (35.45 °N, 107.49 °E) on the central CLP (Fig. 1a) and the locations of the Pacific sites cited for comparisons are illustrated in Fig. 1b. The ^87^Sr/^86^Sr ratios of the 28–45 μm silicate fractions (donated as ^87^Sr/^86^Sr* hereafter) of the loess and paleosol samples show a gradually decreasing trend of about 0.004 since ~2.7 Ma (Supplementary Table S1; Fig. 2). No obvious glacial-interglacial variations in ^87^Sr/^86^Sr* have been observed based on neighboring loess and paleosol samples. The gradually decreasing trend of ^87^Sr/^86^Sr* since ~2.7 Ma is consistent with the records in Jingchuan and Lingtai sections^14^,^25^,^26^ (Fig. 3). The mean value of ^87^Sr/^86^Sr* in this study (0.718978, n = 97) is slightly lower than that of Lingtai site (0.720292, n = 10) by Chen and Li^14^ based on the same grain size fraction. As expected, the mean value of ^87^Sr/^86^Sr* in this study (0.718978, n = 97) is about 0.006 lower than that of the <20 μm silicate fractions of the Jingchuan section (0.724730, n = 66)^25^ and is 0.002 lower than that of the bulk silicate fractions of the Lingtai section (0.721130, n = 43)^26^. Opposite trends of ^87^Sr/^86^Sr ratio between the Xifeng section and the Pacific cores have been observed during 3 and 0.8 Ma (Fig. 1b and Fig. 4). However, the CLP records and Pacific cores show similar decreasing trend of the ^87^Sr/^86^Sr ratio of Asian dust since 0.8 Ma (Fig. 4a and Fig. 4b). The ε_Nd_ value of Xifeng section shows an increasing trend by 1.5ε unit since ~2.7 Ma (Supplementary Table S2; Fig. 2).

## Discussion

The limited variations of ^87^Sr/^86^Sr* between the neighboring loess and paleosol layers (Fig. 2) imply an unchanged eolian source on the CLP during the glacial-interglacial cycles. However, it may be argued that ^87^Sr/^86^Sr* is not sensitive enough to reflect the subtle source changes and the influence of source shifts on ^87^Sr/^86^Sr* is offset by the effect of grain size changes (Fig. 4). We think such possibilities are very unlikely since potential source shift to the Gobi Altay Mountains^17^ would largely decrease ^87^Sr/^86^Sr* due to the low ^87^Sr/^86^Sr ratio of Gobi materials^11^,^13^,^14^,^20^. The possible increasing grain size in the 28–45 μm fraction during glacial times^30^,^31^ will decrease the ^87^Sr/^86^Sr ratio.

It has been shown that the ^87^Sr/^86^Sr ratio of the clay particles is about 0.006 higher than that of other grain size fractions^11^, but the maximum variation of grain size would only introduce less than 0.001 change in the ^87^Sr/^86^Sr ratios of bulk silicate^14^,^20^. Thus, the observed 0.004 shift of the ^87^Sr/^86^Sr* over the past 2.7 Ma (Fig. 2) may not introduced by sorting process but mainly reflect source change. The primary control of source shift on ^87^Sr/^86^Sr* is also supported by the long term shift in ε_Nd_ values (Fig. 2). Unlike Sr isotope, Nd isotope has been commonly used as a robust source tracer with minimal effects from mineral sorting^27^. The negative correlation between Nd and Sr isotopes lies on the binary mixing line between the Gobi Altay Mountains and the Qilian Mountains (Fig. 3), confirming binary source evolution of the eolian deposits on the CLP^13^,^14^,^19^. Thus, the gradual decreasing ^87^Sr/^86^Sr* and increasing ε_Nd_ values reflect a gradual source shift of the CLP from the Qilian Mountains to the Gobi Altay Mountains since ~2.7 Ma (Fig. 3).

The source shift of eolian dust on the CLP over the past ~2.7 Ma might be related to tectonic and climatic changes. It has been shown that the CLP receives eolian dust mainly from the Alxa arid lands by prevailing near surface winds^13^,^14^ (Fig. 1a). However, Alxa arid lands only act a sediment holder rather than a producer. It receives materials mainly from the Gobi Altay Mountains and the Qilian Mountains through fluvial systems^13^. Mountain processes are the most important mechanisms that produce the silt particle of the loess deposits^32^. Mountain erosion is a strong function of relief^33^. The differential uplift history between the Qilian Mountains and the Gobi Altay Mountains may have modulated the relative contribution of debris from the two mountains to the CLP. Considering a relative stable Gobi Altay Mountains, the progressive uplift of North Tibetan Plateau has been inferred to explain the decreasing ε_Nd_ values of Asian dust since the middle Miocene^13^. However, the evidence of late Pliocene uplift of the Tibetan Plateau is controversial^34^. The uplift of the Gobi Altay Mountains since 5 ± 3 Ma^35^ may increase the relative material contribution of the Gobi materials to the Alxa arid lands, and finally the CLP.

The decreasing trend of ^87^Sr/^86^Sr* over the past ~2.7 Ma also seems to match the gradual cooling trend of global climate or growth of Northern Hemisphere glaciations as reflected by the oxygen isotope of benthic foraminifera^36^ (Fig. 2), implying control of climate change on the source shift. Climate change may modulate the eolian source on the CLP by several means. Global cooling is normally accompanied with the drop of snow line and growth of mountain glacial. Glaciation is one of the most efficient ways of physical erosion^37^. Thus, the glacial on the Gobi Altay Mountains produced more materials to the Alxa arid lands. Strengthened Siberia High and thus Asian winter monsoon^7^,^30^,^31^ in response to global cooling would transport more materials^38^ from the Gobi Altay Mountains to the Alxa arid lands (Fig. 4a and Fig. 1a).

The source shift of eolian dust on the CLP might be reflected in the Pacific sediments. Previous studies indicated that the eolian dust in the north central Pacific sediments is mainly derived from the arid lands of Asian interior through westerly winds while the dust deposited in circum-Pacific regions is dominated by volcanic ash^39^. Taklimakan desert in northwest China is suggested to be one of the most important sources for long-range eolian dust transport by westerly jet stream^40^,^41^,^42^. Occasionally, Gobi dust can also be lifted into the middle troposphere and transport to East Asia and north Pacific Oceans^43^ (Fig. 1b).

The pacific sediments show very different evolutionary patterns of Sr isotopic composition compared to the loess on the CLP (Fig. 4a and Fig. 4b). It has been noticed that the north central Pacific sediments are the mixtures of eolian dust from Asian interior arid areas with more radiogenic Sr isotopic composition and volcanic ash with less radiogenic Sr isotopic composition^29^,^39^. Considering a relatively constant volcanic activity over the past 3 Ma^44^, the increasing ^87^Sr/^86^Sr ratios of pacific sediments between 3 and ~0.8 Ma might be caused by increasing Asian dust flux in response to aridification of Asian interior since ~2.7 Ma (Fig. 4b). The decreasing ^87^Sr/^86^Sr ratios since ~0.8 Ma may attribute to the increasing addition of the Gobi dust because contribution of Asian dust dominated the eolian deposits during this period and thus the ^87^Sr/^86^Sr ratio is not sensitive to the changing relative contribution of Asian dust and volcanic ash. The Earth’s climate changed fundamentally after middle Pleistocene transition with the dominant periodicity of glacial cycles shifting from 41 ka to 100 ka^45^. The full glacial climate after middle Pleistocene transition strengthened winter monsoon, which transported more materials from the Gobi Altay Mountains with lower ^87^Sr/^86^Sr ratios to the Pacific Oceans. The increasing eolian flux in the Pacific core V21–146^46^ since ~0.5 Ma and ODP site 885/886^47^ since ~0.8 Ma may have conformed to this climate evolution (Fig. 1b and Fig. 4b).

## Methods

The eolian deposits at the Xifeng site consist of tens of loess and paleosol alternations deposited over the past ~2.7 Ma and the red-clay formation aged from ~6.2 Ma to ~2.7 Ma. The chronology of loess and paleosol deposits have been well constrained by magnetostratigraphy^48^ as well as orbital tuning based on climate proxies of grain size and magnetic susceptibility^30^.

The 97 samples for Sr isotopic analysis are selected based on magnetic susceptibility (Fig. 2). Paleosol layers are characterized by high magnetic susceptibility due to the enhanced pedogenesis during the warm and wet interglacial period while loess layers of low magnetic susceptibility are product of glacial climate^49^. Both samples of high and low magnetic susceptibility are selected for most of the loess and paleosol alternations. The purpose of this sampling strategy is two fold. The neighboring loess layer and paleosol layer have very different grain sizes. Such difference is even larger than the long-term shift of grain size over the past 2.7 Ma^50^. Thus, the samples of neighboring loess and paleosol may help to examine if the restricting 28–45 μm grain-size would eliminate the effect of grain size on the ^87^Sr/^86^Sr signal. Second, the glacial loess may have different eolian source compared to the interglacial paleosol^22^. Thus, the sampling strategy could also eliminate possible bias of the samples to loess or paleosol layers, which enables us to detect the long-term source shift over the past ~2.7 Ma.

To remove carbonate fraction, the selected samples were dissolved in diluted acetic acid (0.5 mol/L) after Chen *et al.*^11^ in the ultrasonic bath for about 10 minutes. Then, the remaining silicate fractions were sieved to obtain 28–45 μm grain size fraction. The extracted 28–45 μm silicate fractions were digested in a mixture of HNO_3_+HF solution. The Sr and Nd elements in the digested solution were then purified using standard ion exchange techniques. The determination of Sr and Nd isotopes were preformed on a Neptune plus Multi-Collector Inductively Coupled Plasma Mass Spectrometer (MC-ICP-MS) at the Department of Earth Sciences, Nanjing University. Instrumental bias was corrected to ^86^Sr/^88^Sr of 0.1194 and ^146^Nd/^144^Nd of 0.7219, respectively. The Sr standard SRM987 and Nd standard JMCNd_2_O_3_ were periodically measured to check the reproducibility and accuracy of isotopic analyses with mean ^87^Sr/^86^Sr ratio of 0.7102387 ± 42 (external standard deviation, n = 10) and mean ^143^Nd/^144^Nd ratio of 0.5120997 ± 15, respectively. Epsilon Nd values (ε_Nd_) were calculated using chondritic values of ^143^Nd/^144^Nd = 0.512638^51^. The analytical results and samples information are listed in Supplementary Table S1 and Supplementary Table S2.

## Additional Information

**How to cite this article**: Zhang, W. *et al.* Shifting material source of Chinese loess since ~ 2.7 Ma reflected by Sr isotopic composition. *Sci. Rep.* **5,** 10235; doi: 10.1038/srep10235 (2015).

## Supplementary Material

Supporting Information
