# Supplementary material for: Shifting material source of Chinese loess since ~2.7 Ma reflected by Sr isotopic composition
Source: Sci Rep. 2015 May 21;5:10235. doi: 10.1038/srep10235 (PMC4650810; doi:10.1038/srep10235)
Supplement: Supporting Information [file srep10235-s1.doc]

**Supplementary Information**

**Shifting material source of Chinese loess since ~ 2.7 Ma reflected by Sr isotopic composition**

Wenfang Zhang^1*^, Jun Chen^1*^, Gaojun Li^1*^

^1^MOE Key Laboratory of Surficial Geochemistry, Department of Earth Sciences, Nanjing University, 163 Xianlindadao, Nanjing 210046, China.

^*^Corresponding E-mail: nic_langdi@163.com; chenjun@nju.edu.cn; ligaojun@nju.edu.cn

# Supplementary Tables

**Table S1**: The depth, age, and the Sr isotopic compostions of the samples collected in Xifeng section.

| **Depth(m)** | **Age(kyr) ^a^** | **^87^Sr/^86^Sr** | **SE** |
| --- | --- | --- | --- |
| 0.05 | 0.00 | 0.717360 | 10 |
| 0.45 | 2.19 | 0.717157 | 10 |
| 0.65 | 4.09 | 0.717371 | 10 |
| 0.90 | 6.79 | 0.717510 | 13 |
| 1.75 | 13.06 | 0.717462 | 8 |
| 2.40 | 20.63 | 0.717356 | 12 |
| 4.05 | 25.16 | 0.717569 | 12 |
| 5.25 | 29.79 | 0.717458 | 8 |
| 6.70 | 37.78 | 0.717627 | 8 |
| 7.90 | 43.77 | 0.717611 | 8 |
| 10.05 | 60.01 | 0.717561 | 7 |
| 11.55 | 77.97 | 0.718053 | 11 |
| 12.00 | 94.40 | 0.717970 | 9 |
| 13.80 | 125.25 | 0.717435 | 10 |
| 14.60 | 129.18 | 0.717236 | 11 |
| 17.25 | 152.00 | 0.717415 | 8 |
| 18.95 | 159.43 | 0.717494 | 7 |
| 21.90 | 179.50 | 0.718073 | 12 |
| 24.00 | 204.08 | 0.718610 | 11 |
| 24.60 | 215.46 | 0.718248 | 12 |
| 25.25 | 223.43 | 0.718002 | 7 |
| 26.75 | 239.65 | 0.718244 | 12 |
| 29.15 | 272.84 | 0.718455 | 10 |
| 31.30 | 298.88 | 0.718930 | 10 |
| 33.90 | 340.19 | 0.718059 | 10 |
| 36.80 | 357.47 | 0.718943 | 13 |
| 38.70 | 403.36 | 0.718576 | 14 |
| 40.15 | 426.55 | 0.717904 | 12 |
| 40.55 | 428.24 | 0.717614 | 9 |
| 40.95 | 429.97 | 0.717514 | 6 |
| 43.80 | 445.96 | 0.718974 | 14 |
| 46.55 | 472.43 | 0.719312 | 7 |
| 46.85 | 475.01 | 0.719367 | 8 |
| 48.15 | 494.17 | 0.718861 | 8 |
| 48.60 | 500.79 | 0.718743 | 12 |
| 50.45 | 545.24 | 0.718449 | 9 |
| 52.45 | 612.73 | 0.717945 | 9 |
| 53.95 | 627.59 | 0.718112 | 9 |
| 54.65 | 632.23 | 0.718541 | 11 |
| 56.10 | 646.12 | 0.718509 | 10 |
| 58.80 | 666.40 | 0.718760 | 7 |
| 60.70 | 694.08 | 0.718782 | 10 |
| 60.90 | 700.82 | 0.718678 | 9 |
| 61.30 | 711.20 | 0.718651 | 10 |
| 62.05 | 717.01 | 0.718659 | 11 |
| 63.60 | 728.80 | 0.718974 | 8 |
| 64.75 | 751.77 | 0.718881 | 11 |
| 67.00 | 770.69 | 0.718765 | 10 |
| 68.70 | 794.26 | 0.718807 | 10 |
| 71.50 | 832.24 | 0.718649 | 10 |
| 73.10 | 852.72 | 0.718692 | 9 |
| 75.65 | 873.50 | 0.718969 | 10 |
| 78.20 | 891.49 | 0.719120 | 11 |
| 79.30 | 905.72 | 0.719239 | 8 |
| 82.20 | 922.06 | 0.719287 | 9 |
| 83.80 | 950.20 | 0.719327 | 11 |
| 84.85 | 961.95 | 0.719485 | 9 |
| 89.15 | 1030.98 | 0.719719 | 12 |
| 91.80 | 1066.25 | 0.719322 | 12 |
| 93.15 | 1079.10 | 0.719586 | 11 |
| 94.80 | 1094.59 | 0.719022 | 12 |
| 99.00 | 1177.20 | 0.719433 | 12 |
| 100.75 | 1214.53 | 0.719052 | 19 |
| 102.45 | 1244.09 | 0.718750 | 11 |
| 104.10 | 1252.92 | 0.719246 | 18 |
| 106.65 | 1275.52 | 0.719538 | 90 |
| 108.40 | 1299.15 | 0.719497 | 10 |
| 110.35 | 1320.86 | 0.720167 | 9 |
| 110.80 | 1328.73 | 0.719991 | 11 |
| 111.65 | 1341.80 | 0.720076 | 11 |
| 112.40 | 1353.59 | 0.719480 | 10 |
| 113.60 | 1379.65 | 0.720029 | 9 |
| 114.40 | 1384.65 | 0.719865 | 13 |
| 115.05 | 1388.71 | 0.719443 | 10 |
| 115.75 | 1412.63 | 0.719694 | 11 |
| 116.60 | 1444.21 | 0.719803 | 8 |
| 118.05 | 1462.15 | 0.719630 | 10 |
| 118.85 | 1475.00 | 0.719638 | 9 |
| 120.15 | 1498.51 | 0.719932 | 7 |
| 125.80 | 1646.48 | 0.720199 | 10 |
| 127.10 | 1650.38 | 0.719708 | 9 |
| 133.70 | 1714.27 | 0.719590 | 9 |
| 139.45 | 1829.41 | 0.720101 | 10 |
| 143.25 | 1909.36 | 0.720075 | 10 |
| 146.05 | 1963.77 | 0.720238 | 9 |
| 153.95 | 2082.92 | 0.720294 | 11 |
| 158.45 | 2167.15 | 0.720324 | 13 |
| 159.20 | 2180.37 | 0.720699 | 9 |
| 159.90 | 2193.61 | 0.720566 | 8 |
| 162.65 | 2300.74 | 0.720603 | 9 |
| 163.15 | 2322.64 | 0.721111 | 8 |
| 164.00 | 2341.10 | 0.720585 | 8 |
| 166.95 | 2434.04 | 0.719674 | 11 |
| 168.25 | 2457.09 | 0.720572 | 9 |
| 170.10 | 2503.41 | 0.720860 | 11 |
| 172.05 | 2561.88 | 0.719938 | 10 |
| 177.50 | 2727.37 | 0.721459 | 11 |

^a^ The age is interpolated from Sun et al.^1^ and Sun et al.^2^.

**Table S2**: The depth, age, and the Nd isotopic compostions of the samples collected in Xifeng section.

| **Depth(m)** | **Age (kyr) ^a^** | **^143^Nd/^144^Nd** | **SE** | **ε_Nd_^d^** |
| --- | --- | --- | --- | --- |
| 0.65 | 4.09 | 0.512066 | 3 | -11.2 |
| 2.4 | 20.63 | 0.512126 | 7 | -10.0 |
| 5.25 | 29.79 | 0.512113 | 5 | -10.2 |
| 10.05 | 60.01 | 0.512090 | 6 | -10.7 |
| 12.00 | 94.40 | 0.512114 | 3 | -10.2 |
| 14.60 | 129.18 | 0.512124 | 6 | -10.0 |
| 18.95 | 159.43 | 0.512103 | 7 | -10.4 |
| 24.6 | 215.46 | 0.512091 | 5 | -10.7 |
| 26.75 | 239.65 | 0.512094 | 5 | -10.6 |
| 31.3 | 298.88 | 0.512085 | 8 | -10.8 |
| 36.8 | 357.47 | 0.512096 | 5 | -10.6 |
| 40.55 | 428.24 | 0.512074 | 5 | -11.0 |
| 46.55 | 472.43 | 0.512102 | 7 | -10.5 |
| 48.6 | 500.79 | 0.512109 | 5 | -10.3 |
| 52.45 | 612.73 | 0.512094 | 6 | -10.6 |
| 60.7 | 694.08 | 0.512096 | 6 | -10.6 |
| 62.05 | 717.01 | 0.512093 | 4 | -10.6 |
| 63.6 | 728.80 | 0.512076 | 5 | -11.0 |
| 67 | 770.69 | 0.512097 | 4 | -10.6 |
| 71.5 | 832.24 | 0.512087 | 3 | -10.7 |
| 73.1 | 852.72 | 0.512070 | 5 | -11.1 |
| 78.2 | 891.49 | 0.512081 | 6 | -10.9 |
| 82.2 | 922.06 | 0.512071 | 8 | -11.1 |
| 84.85 | 961.95 | 0.512069 | 2 | -11.1 |
| 91.8 | 1066.25 | 0.512103 | 5 | -10.4 |
| 94.8 | 1094.59 | 0.512082 | 3 | -10.8 |
| 99 | 1177.20 | 0.512083 | 4 | -10.8 |
| 102.45 | 1244.09 | 0.512076 | 9 | -11.0 |
| 106.65 | 1275.52 | 0.512076 | 3 | -11.0 |
| 108.4 | 1299.15 | 0.512056 | 3 | -11.4 |
| 110.8 | 1328.73 | 0.512048 | 4 | -11.5 |
| 112.4 | 1353.59 | 0.512079 | 6 | -10.9 |
| 114.4 | 1384.65 | 0.512065 | 4 | -11.2 |
| 115.75 | 1412.63 | 0.512046 | 5 | -11.5 |
| 118.05 | 1462.15 | 0.512065 | 3 | -11.2 |
| 120.15 | 1498.51 | 0.512077 | 4 | -10.9 |
| 125.8 | 1646.48 | 0.512075 | 3 | -11.0 |
| 133.7 | 1714.27 | 0.512076 | 9 | -11.0 |
| 143.25 | 1909.36 | 0.512067 | 6 | -11.1 |
| 153.95 | 2082.92 | 0.512102 | 6 | -10.5 |
| 158.45 | 2167.15 | 0.512048 | 7 | -11.5 |
| 159.9 | 2193.61 | 0.512047 | 3 | -11.5 |
| 163.15 | 2322.64 | 0.512063 | 2 | -11.2 |
| 166.95 | 2434.04 | 0.512076 | 9 | -11.0 |
| 170.1 | 2503.41 | 0.512062 | 4 | -11.2 |
| 177.5 | 2727.37 | 0.512049 | 5 | -11.5 |

^a^ The age is same as in Table S1; ^b^ ε_Nd_ values are expressed relative to chondrite values of ^143^Nd/^144^Nd=0.512638^3^: ε_Nd_ = (^143^Nd/^144^Nd−0.512638) /0.512638 × 10000.

1. Sun, D. H., Shaw, J., An, Z. S., Cheng, M. Y. & Yue, L. P. Magnetostratigraphy and paleoclimatic interpretation of a continuous 7.2Ma Late Cenozoic eolian sediments from the Chinese Loess Plateau. *Geophys Res Lett* **25**, 85-88 (1998).
2. Sun, Y. B., Clemens, S. C., An, Z. S. & Yu, Z. W. Astronomical timescale and palaeoclimatic implication of stacked 3.6-Myr monsoon records from the Chinese Loess Plateau. *Quatern. Sci. Rev.* **25**, 33-48 (2006).
3. Jacobsen, S. B. & Wasserburg, G. J. Sm-Nd isotopic evolution of chondrites. *Earth Planet Sc Lett* **50**, 139-155, doi:<http://dx.doi.org/10.1016/0012-821X(80)90125-9> (1980).
